# Role of the EF-hand and coiled-coil domains of human Rab44 in localisation and organelle formation

**DOI:** 10.1038/s41598-020-75897-7

**Published:** 2020-11-05

**Authors:** Kohei Ogawa, Tomoko Kadowaki, Mitsuko Tokuhisa, Yu Yamaguchi, Masahiro Umeda, Takayuki Tsukuba

**Affiliations:** 1 Department of Frontier Oral Science, Graduate School of Biomedical Sciences, Nagasaki University, Sakamoto 1-7-1, Nagasaki, 852-8588 Japan; 2 Department of Dental Pharmacology, Graduate School of Biomedical Sciences, Nagasaki University, Sakamoto 1-7-1, Nagasaki, 852-8588 Japan; 3 Department of Clinical Oral Oncology, Graduate School of Biomedical Sciences, Nagasaki University, Sakamoto 1-7-1, Nagasaki, 852-8588 Japan

**Keywords:** Biochemistry, Cell biology

## Abstract

Rab44 is a large Rab GTPase that contains an amino-terminal EF-hand domain, a coiled-coil domain, and a carboxyl-terminal Rab GTPase domain. However, the roles of the EF-hand and coiled-coil domains remain unclear. Here, we constructed various deletion and point mutants of human Rab44. When overexpressed in HeLa cells, the wild-type Rab44 (hWT) formed ring-like structures, and partially localised to lysosomes. The dominant negative mutant, hT847N, localised to lysosomes and the cytosol, while the constitutively active mutant, hQ892L, formed ring-like structures, and partially localised to the plasma membrane and nuclei. The hΔEF, hΔcoil, and h826-1021 mutants also formed ring-like structures; however, their localisation patterns differed from hWT. Analysis of live imaging with LysoTracker revealed that the size of LysoTracker-positive vesicles was altered by all other mutations than the hC1019A and hΔEF. Treatment with ionomycin, a Ca^2+^ ionophore, induced the translocation of hWT and hΔcoil into the plasma membrane and cytosol, but had no effect on the localisation of the hΔEF and h826-1021 mutants. Thus, the EF- hand domain is likely required for the partial translocation of Rab44 to the plasma membrane and cytosol following transient Ca^2+^ influx, and the coiled-coil domain appears to be important for localisation and organelle formation.

## Introduction

Intracellular membrane trafficking coordinates complex network systems that precisely regulate various membrane-bound organelles in cells^1,2^. Dynamic intracellular movements such as vesicle transport, membrane fission, tethering, docking, and fusion events are centered on Rab GTPases acting as critical regulators^3,4^. Rab GTPases translocate between the cytosol and membranes by undergoing conformational changes that are strictly controlled by two important regulators: the guanine nucleotide exchange factor (GEF) and GTPase activating protein (GAP)^5,6^. GEFs convert the inactive GDP-bound Rab protein to the active GTP-bound form on membranes, while GAPs catalyse the active GTP-bound Rab protein to the inactive GDP-bound form^7,8^. In addition, lipid modification is also an important characteristic of Rab GTPases. For the membrane binding functions, Rab GTPases contain one or two cysteine residues in the carboxy-terminus (C-terminus) that undergo post-translational modification through prenylation^9,10^. To date, approximately 70 members of Rab GTPases have been identified in mammals^11,12^.

Among these, Rabs 1–43 are small Rab GTPases with a molecular weight of about 20–30 kDa^13^. In contrast, Rab44, Rab45, and CRACR2A (also known as Rab46) are large Rab GTPases that contain a few additional domains including EF-hand and coiled-coil domains in addition to the Rab-GTPase domain^14–16^. Rab44 was recently identified as a protein that is upregulated during osteoclast differentiation by our research group^17^. Using knockdown (with small interfering RNA) and overexpression systems, we showed that Rab44 negatively regulates osteoclast differentiation by modulating intracellular calcium levels following NFATc1 activation. Comparing the sequence of the human and mouse Rab44 reveals that both Rab44 proteins contain EF-hand, coiled-coil, and Rab-GTPase domains. Initially, mouse Rab44 was identified as only a short form lacking the EF-hand domain^17^. However, our recent studies reported that mouse mast cells and bone-marrow cells have two isoforms: the long form including the EF-hand domain and the short form lacking this domain^18,19^. These findings have raised fundamental questions about the differences in localisation and function of Rab44 between humans and mice. Indeed, our recent study using the murine macrophage cell line, RAW-D cells, showed that mouse Rab44 localised mainly to lysosomes and the Golgi complex^17^. There are currently no studies on the localisation of human Rab44. Moreover, the roles of the amino-terminal EF-hand and coiled-coil domains in the localisation of human Rab44 remain unclear.

In this study, we investigated the mechanisms involved in the localisation of human Rab44 by ectopically expressing various Rab44 mutants in HeLa cells, which are human epithelial cells most often used in intracellular localisation analyses.

## Results

### Ectopic expression and localisation of wild type and mutants of human Rab44 in HeLa cells

**Figure 1:**
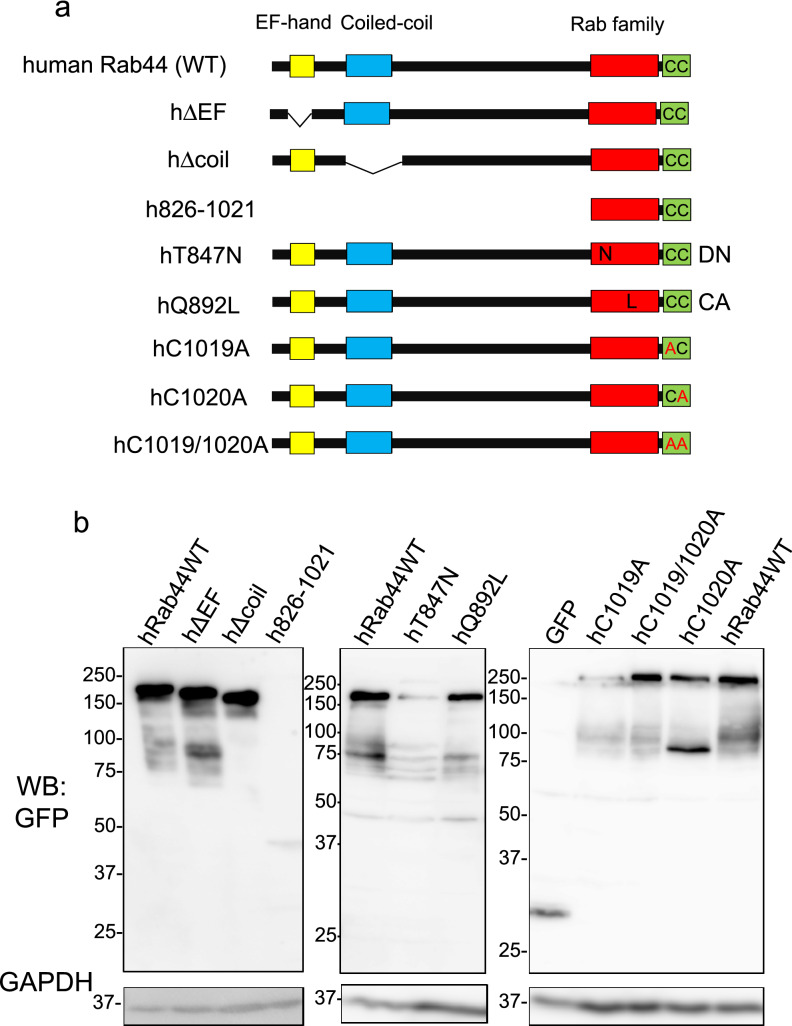
Construction and ectopic expression of wild type and various Rab44 mutants in HeLa cells. (**a**) Schematic representation of wild type and mutant Rab44 expressed in HeLa cells. The EF-hand, coiled-coil, Rab family, and lipidation domains are shown in yellow, blue, red, and green respectively. The recombinant Rab44 proteins were expressed as N-terminal GFP-fusion proteins. (**b**) Western blot analysis of HeLa cell expressing Rab44 and its derivatives.

To investigate the subcellular localisation of human Rab44, we constructed various mutants of human Rab44 by site-directed mutagenesis (Fig. 1a), including wild-type human Rab44 (hWT), an EF-hand motif deletion (hΔEF), a coiled-coil domain deletion (hΔcoil), a Rab domain only (h826-1021), a constitutively active (CA) mutant (hQ892L), a dominantly negative (DN) mutant (hT847N), and C-terminal lipidation site mutants with point mutations at residues 1019 and 1020 (C1019A, C1020A, and C1019A/C1020A). The hWT and mutant constructs were exogenously expressed as N-terminal green fluorescent protein (GFP)-fusion proteins in HeLa cells (Fig. 1a). The protein levels of the mutants in the HeLa cells were confirmed by western blot analysis (Fig. 1b). The expressed proteins were detected at their predicted molecular weights, although the expression levels of h826-1021, hT847N, and C1019A were relatively low.

**Figure 2:**
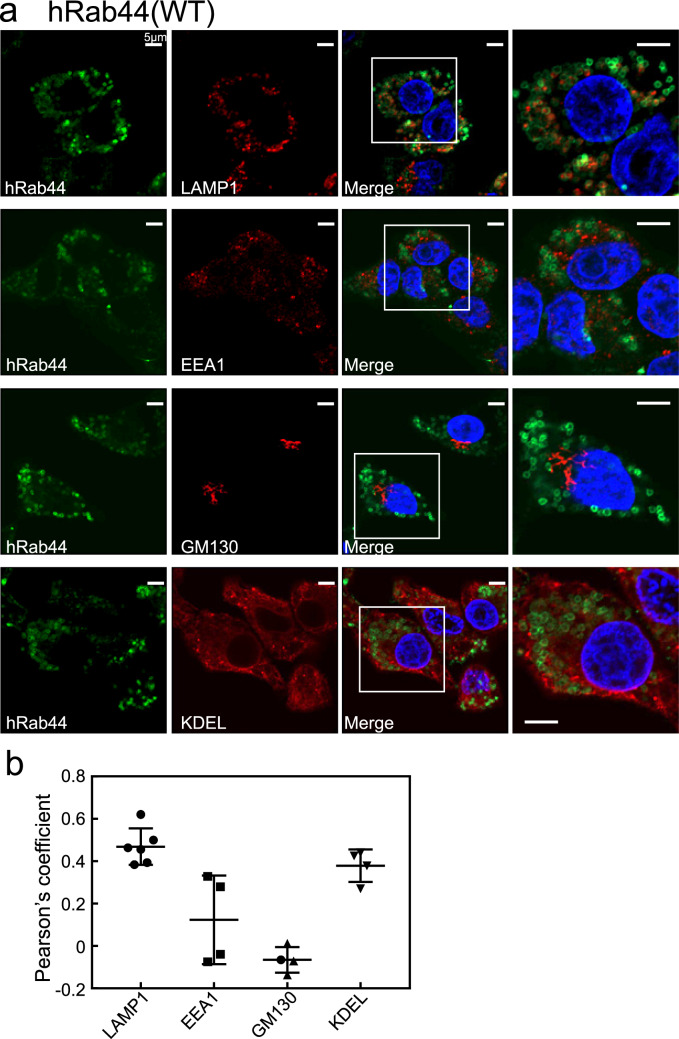
Subcellular localisation of eGFP-hRab44 (hWT) in HeLa cells. (**a**) Confocal laser microscopic analysis of HeLa cells immunofluorescently stained for LAMP1 (marker for late endosomes/lysosomes), EEA1 (marker for early endosomes), GM130 (marker for the Golgi), and KDEL (marker for ER). Bar: 5 μm. (**b**) Quantitative analysis of the ratio of colocalisation of hWT with indicated organelle markers. Data are mean ± SD (n ≥ 4).

The ectopically expressed hWT formed ring-like structures, and partially surrounded the LAMP1-positive lysosomes in HeLa cells (Fig. 2a). However, the hWT hardly merged with EEA1 (a marker for early endosomes)- and GM130 (a marker for the Golgi complex)-positive compartments. (Fig. 2a). Interestingly, the hWT partially merged with KDEL [a marker for endoplasmic reticulum (ER)]-positive compartments. Quantitative analysis of colocalisation between the hWT and these organelle markers is shown in Fig. 2b. We determined that an average score of 0.4 or more is colocalised, 0.2 to 0.4 is partially colocalised, and score of 0.2 or less is not colocalised. Similar determinations were performed in subsequent experiments.

**Figure 3:**
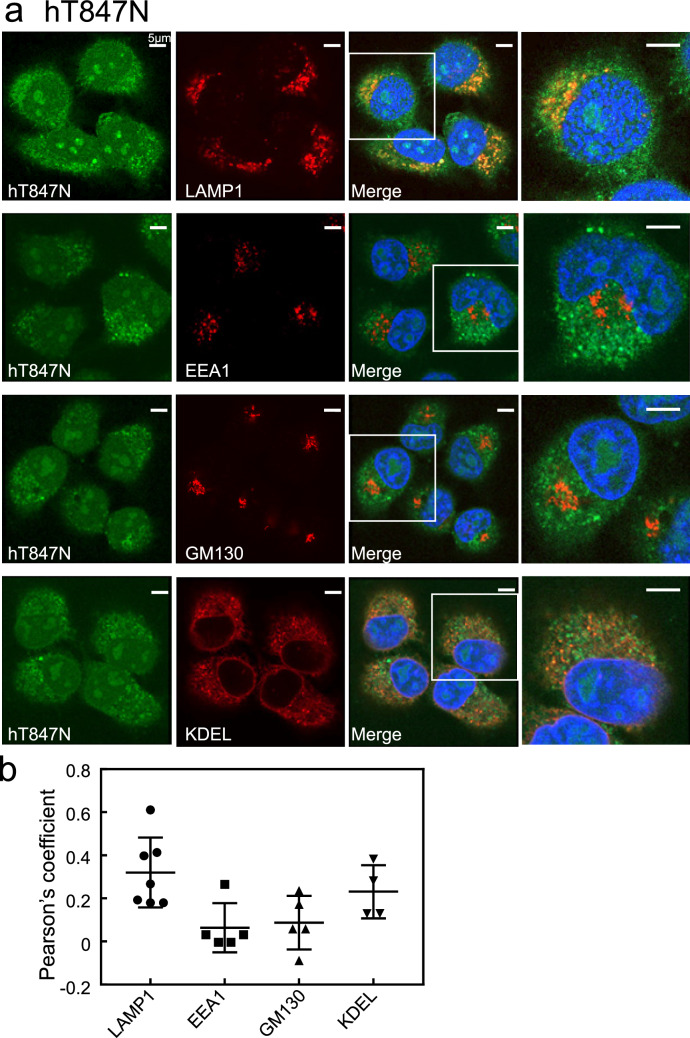

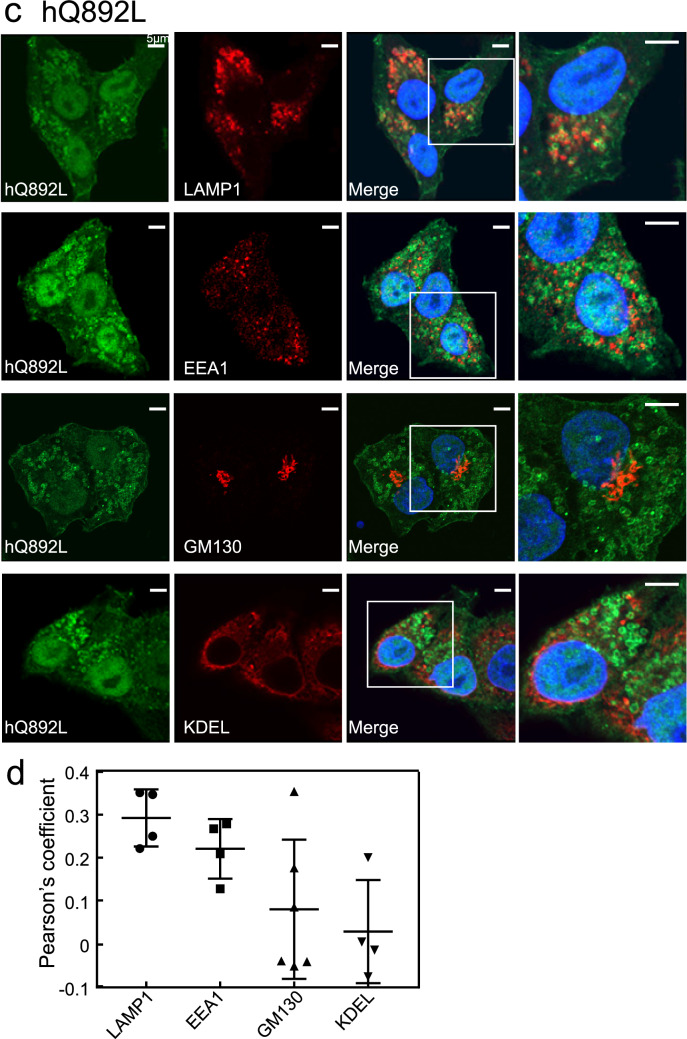

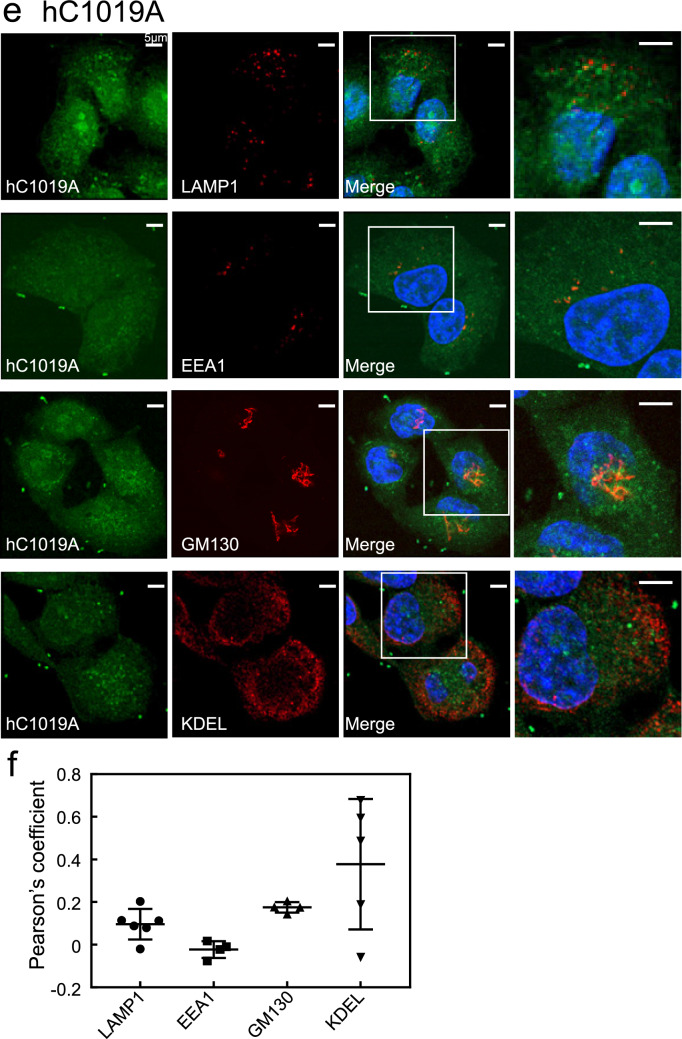

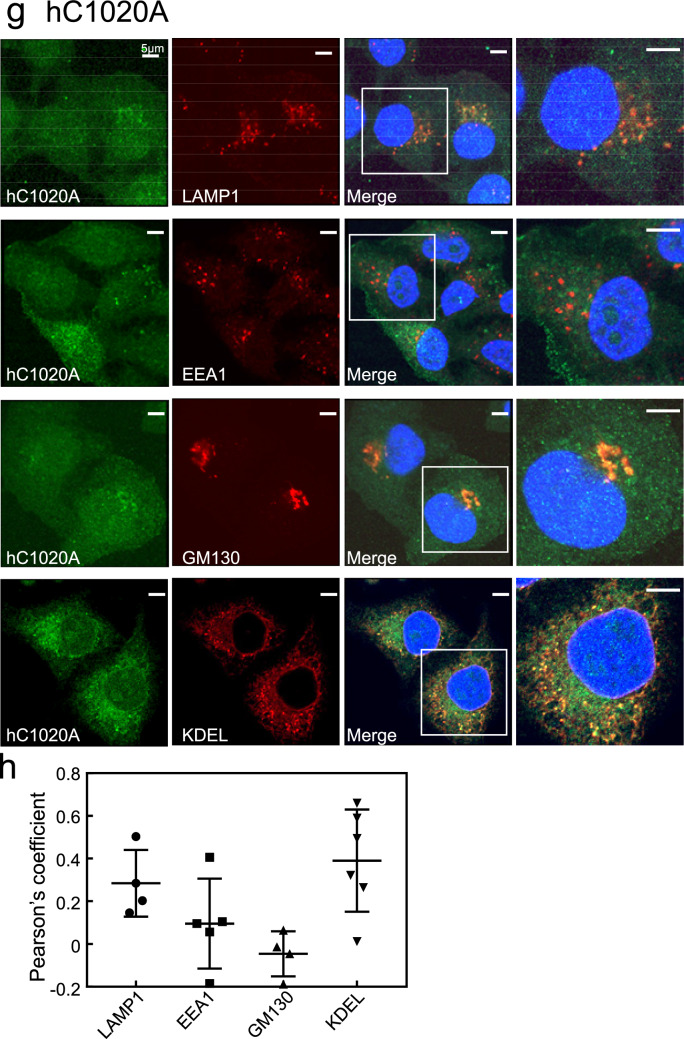

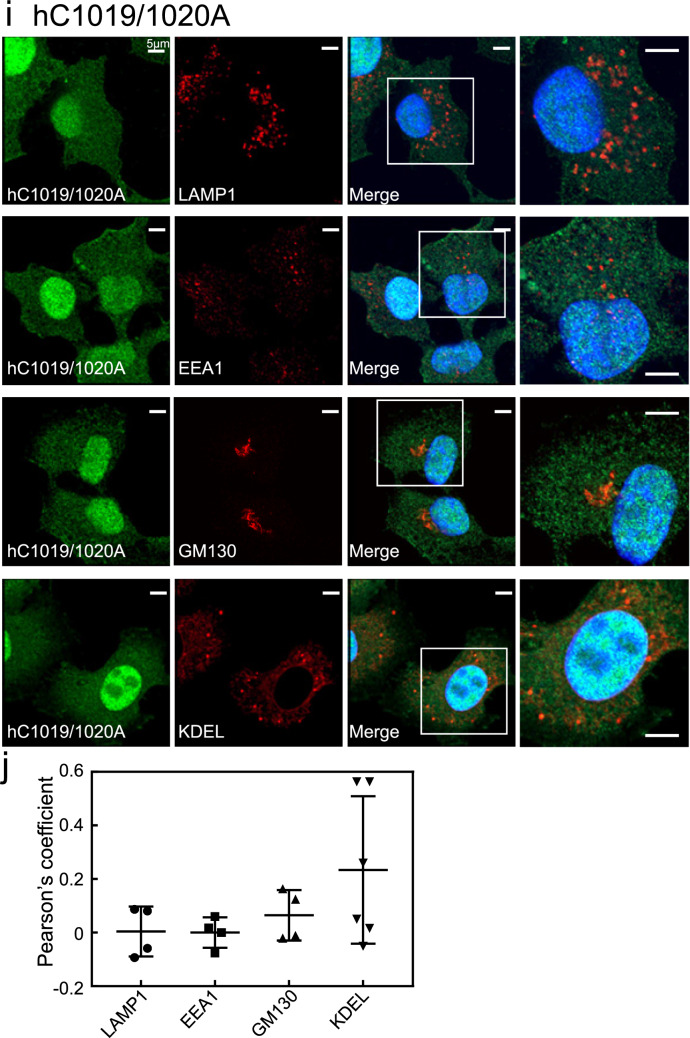
Subcellular localisation of hRab44 mutants in HeLa cells. (**a,b**) eGFP-hT847N (DN) in HeLa cells, (**c,d**) eGFP-hQ892L (CA), (**e,f**) eGFP-hC1019A. (**g,h**) eGFP-hC1020A, and (**i,j**) eGFP-hC1019/1020A. (**a, c, e, g, i**) Confocal laser microscopic analysis of HeLa cells immunofluorescently stained for LAMP1 (marker for late endosomes/lysosomes), EEA1 (marker for early endosomes), GM130 (marker for the Golgi), and KDEL (marker for ER). Bar: 5 μm. (**b, d, f, h, j**) Quantitative analysis of the ratio of colocalisation of these mutants with these organelle markers. Data are mean ± SD (n ≥ 4).

The DN mutant, hT847N, was mostly diffuse, indicating cytosolic localisation, and colocalised partially with LAMP1-positive lysosomes and slightly with the KDEL-positive ER (Fig. 3a). However, hT847N failed to merge with EEA1-positive early endosomes and the GM130-positive Golgi complex (Fig. 3a). Intriguingly, HeLa cells expressing the DN mutant hT847N were rounder in shape than those expressing hWT (see Fig. 2). Quantification of colocalisation between the hT847N and these organelle markers is also shown in Fig. 3b.

The CA mutant, hQ892L, formed ring-like structures and was partially detectable in LAMP1- and EEA1-positive compartments, the plasma membrane, and the nuclei, but hardly detectable in the GM130- and KDEL-positive compartments (Fig. 3c). Of note, in hQ892L-expressing HeLa cells, the shape of the cells and the nuclei were distorted, the cell–cell contact sites were unclear, and hQ892L appeared to promote cell–cell adhesion (Fig. 3c). These results were confirmed by quantitative analysis of colocalisation between the hQ892L and these organelle markers (Fig. 3d).

One of the lipidation-site mutants, hC1019A, was diffusely distributed throughout the cytoplasm, although it partially localised to the KDEL-positive compartments (Fig. 3e). However, the hC1019A mutant was hardly detectable in the LAMP1-, GM130-, and EEA1-positive compartments (Fig. 3e). Consistent with these results, hC1019A colocalisation analysis revealed a colocalisation score of 0.4 with KDEL, and only 0.2 or less with other organelle markers (Fig. 3f).

The other lipidation-site mutant, hC1020A, was also diffusely detected in the cytoplasm, and partially colocalised with LAMP1- and KDEL-positive compartments (Fig. 3g). Indeed, quantitative analysis of colocalisation of hC1020A yielded colocalisation values of 0.2–0.4 with LAMP1 and KDEL (Fig. 3h).

The double mutant hC1019/1020A was also mostly diffusely distributed in the cytoplasm with partial colocalisation with KDEL-positive compartments, and was undetectable in other organelles (Fig. 3i). Quantification of colocalisation between the hC1019/1020A and these organelle markers validated these observations (Fig. 3j).

**Figure 4:**
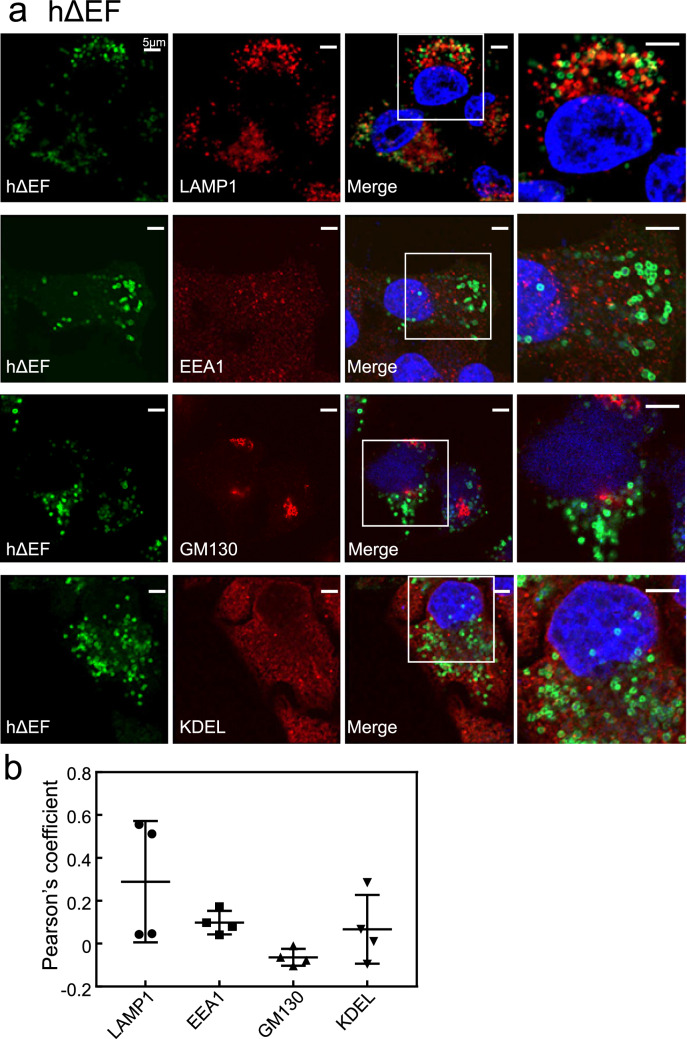

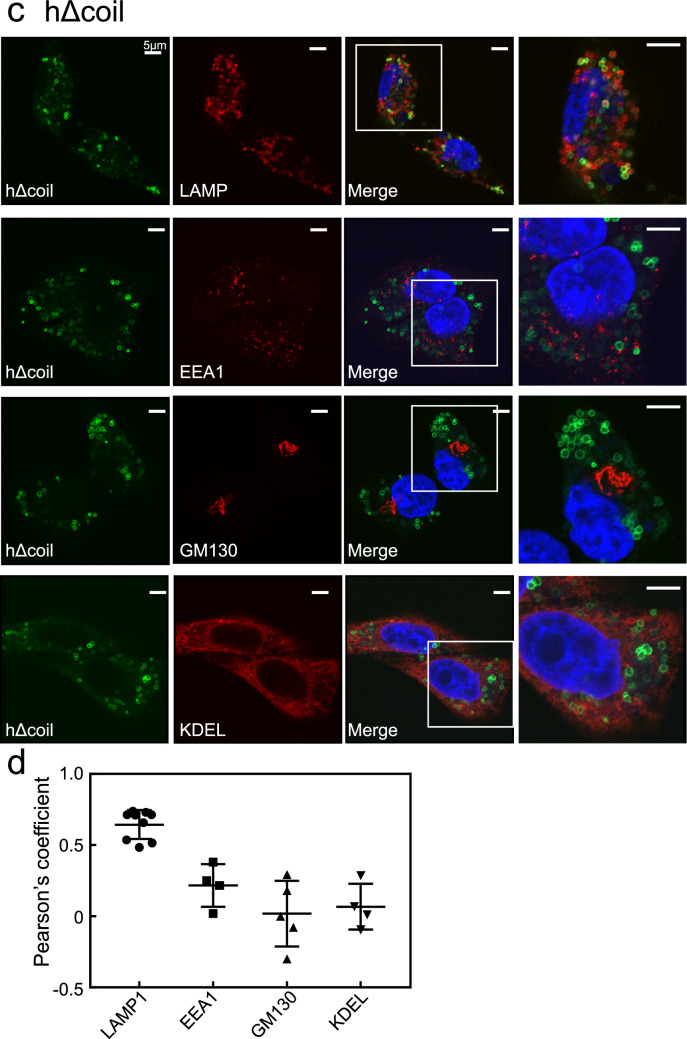

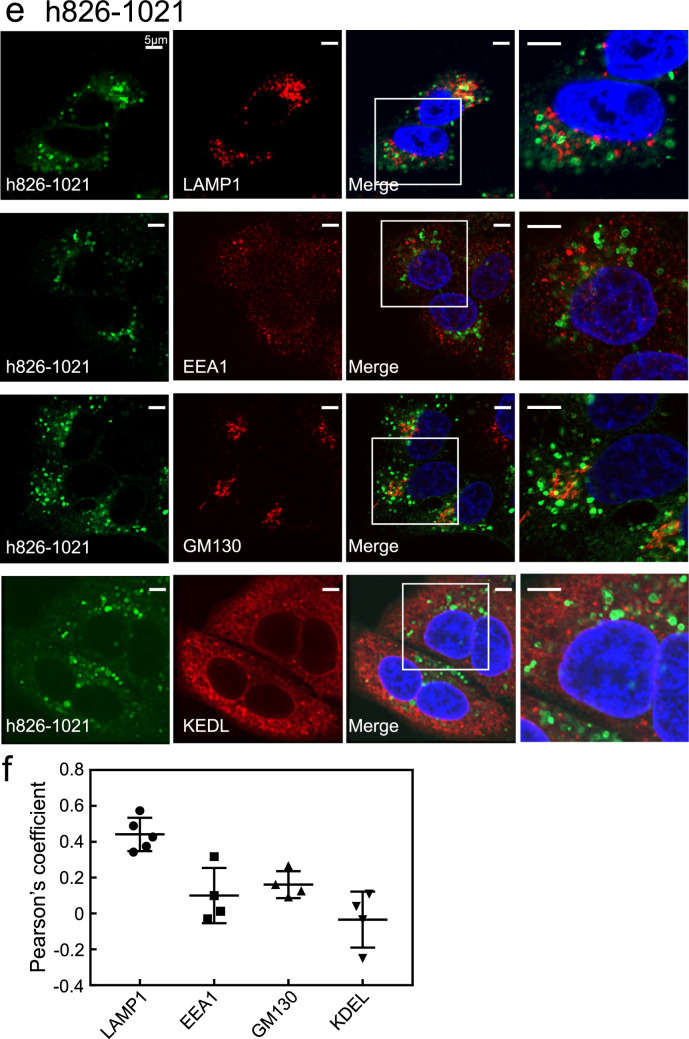
Subcellular localisation of the deletion mutants of hRab44 in HeLa cells. (**a,b**) eGFP-hΔEF, (**c,d**) eGFP-hΔcoil, and (**e,f**) eGFP-h826-1021. (**a, c, e**) Confocal laser microscopic analysis of HeLa cells immunofluorescently stained for LAMP1 (marker for late endosomes/lysosomes), EEA1 (marker for early endosomes), GM130 (marker for the Golgi), and KDEL (marker for ER). Bar: 5 μm. (**b, d, f**) Quantitative analysis of the ratio of colocalisation of these mutants with these organelle markers. Data are mean ± SD (n ≥ 4).

The hΔEF mutant formed ring-like structures, which were partially merged with LAMP1-positive lysosomes (Fig. 4a). The hΔEF mutant did not colocalise with EEA1-, GM130-, or KDEL-positive compartments (Fig. 4a). Quantitative analysis of colocalisation between the hΔEF mutant and these organelle markers indicated its localisation pattern differed from hWT. (Fig. 4b).

The hΔcoil mutant formed ring-like structures and mainly merged with LAMP1-positive lysosomes, but not with EEA1-, GM130-, or KDEL-positive compartments (Fig. 4c). Quantification of the colocalisation of hΔcoil mutant with these organelle markers showed that its localisation pattern differed from hWT (Fig. 4d). The colocalisation score of the hΔcoil mutant with LAMP1-positive lysosomes was higher than that of hWT, and that with KDEL-positive compartment was lower than that of hWT, suggesting that fusion of the hΔcoil mutant with lysosomes is promoted but fusion with the ER is reduced (Fig. 4d).

The h826-1021 mutant mostly formed ring-like structures, although it partially induced vesicle-like structures (Fig. 4e). The h826-1021 mutant colocalised with LAMP1-positive lysosomes, but not with other organelle markers (Fig. 4e), confirmed in quantitative colocalisation analysis (Fig. 4f). Similar to the hΔcoil mutant, the h826-1021 mutant also had a higher colocalisation coefficient with LAMP1-positive lysosomes and a lower one with KDEL-positive compartment compared with hWT (Fig. 4f).

### Effects of human Rab44 and its mutants on formation of LysoTracker-positive compartments in live HeLa cells

**Figure 5:**
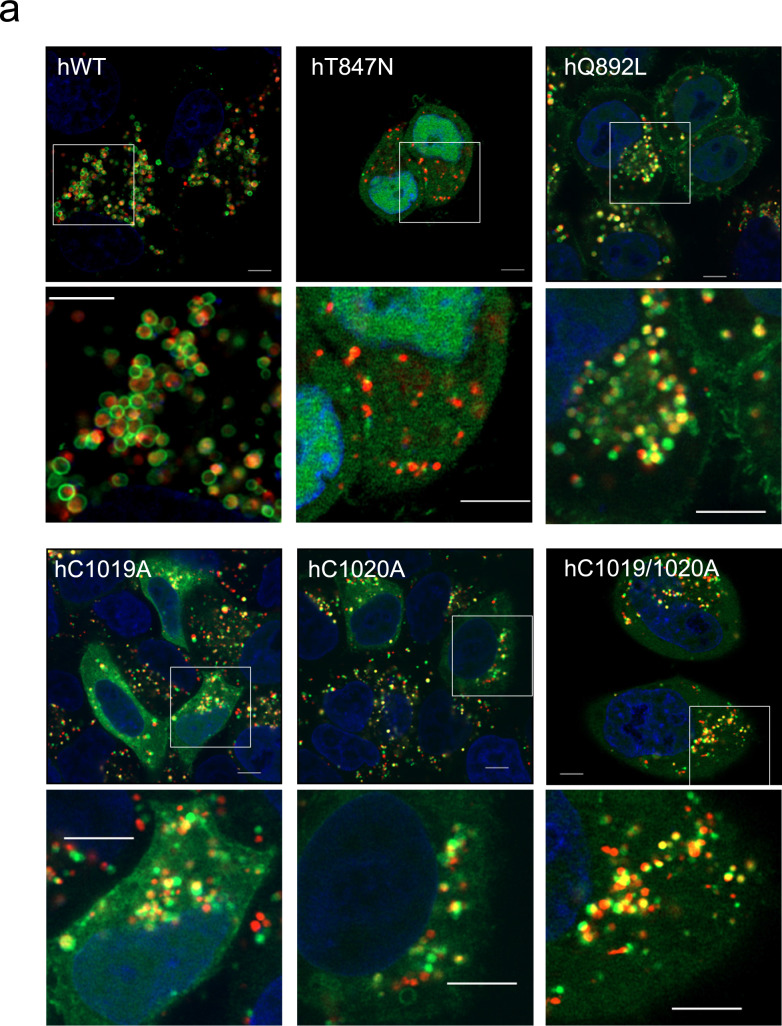

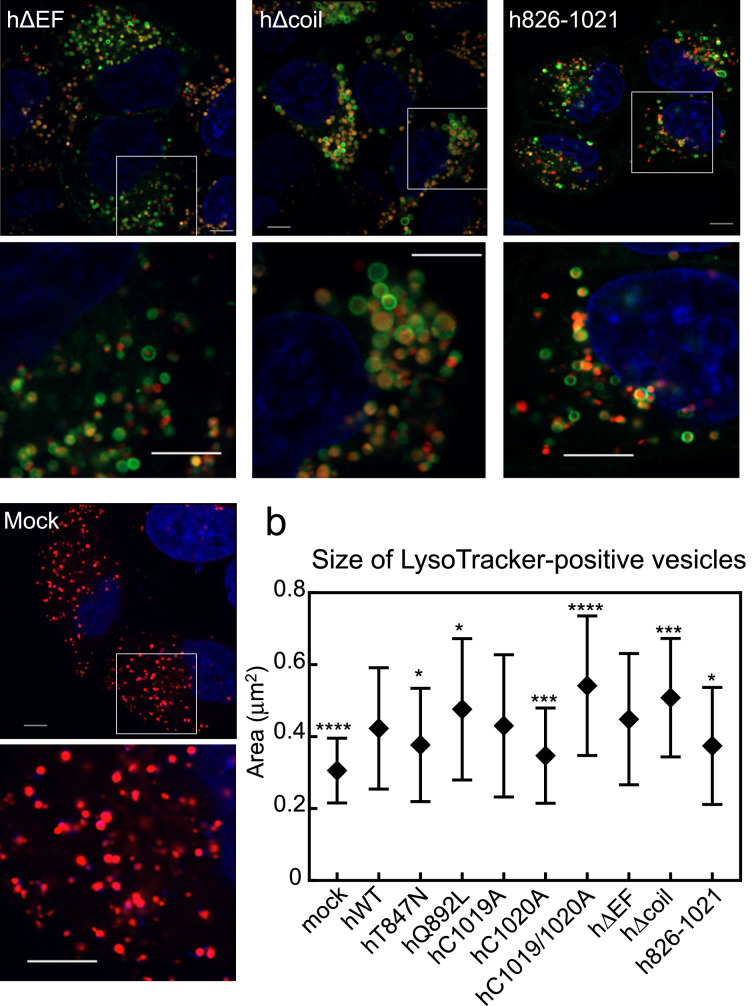
Subcellular localisation of LysoTracker in living HeLa cells expressing hWT and Rab44 mutants (eGFP-hT847N, eGFP-hQ892L, eGFP-hC1019A, eGFP-hC1020A, eGFP-hC1019/1020A, eGFP-hΔEF, eGFP-hΔcoil, and eGFP-h826-1021). (**a**) Confocal laser microscopic analysis of live HeLa cells treated with LysoTracker-Red. Bar: 5 μm. (**b**) Size of LysoTracker-positive vesicles in the live HeLa cells. The area of LysoTracker-positive vesicles was calculated using ImageJ, and the data were subjected to statistical analysis using 1-way ANOVA. Data are mean ± SD (n ≥ 100). The asterisks indicate statistical significance compared to the hWT, *P < 0.05, ***P < 0.001, and ****P < 0.0001.

To investigate the effects of the hWT and mutant constructs on formation of acidic compartments, we measured the size of LysoTracker-positive vesicles in live Hela cells expressing human Rab44 and its mutants. Figure 5a shows the localisation pattern of hWT and its mutants in living cells stained with LysoTracker. Observations in live cells (Fig. 5a) were similar to those in fixed cells, as shown in Figs. 2, 3 and 4.

Quantitative analysis of the size of LysoTracker-positive compartments in live Hela cells are shown in Fig. 5b. hWT induced significantly larger LysoTracker-positive vesicles compared with control (Mock) (Fig. 5b). Interestingly, the DN mutant hT847N induced smaller LysoTracker-positive vesicles than hWT, while the CA mutant hQ892L induced larger LysoTracker-positive vesicles than hWT (Fig. 5b). The size of LysoTracker-positive vesicles was similar between hC1019A- and hWT-expressing cells (Fig. 5b). However, LysoTracker-positive vesicles were smaller in the C1020A-expressing cells and larger in C1019/1020A-expressing cells compared to those in hWT-expressing cells (Fig. 5b). Moreover, the hΔEF mutant yielded similar LysoTracker-positive vesicles to hWT. However, LysoTracker-positive vesicles were larger in hΔcoil-expressing cells and smaller in h826-1021-expressing cells compared to those in hWT-expressing cells (Fig. 5b). These results indicate that the size of LysoTracker-positive vesicles is altered by Rab44 mutations (Fig. 5b).

### Comparison of colocalisation of the human Rab44 mutants with LAMP1 and LysoTracker

**Figure 6:**
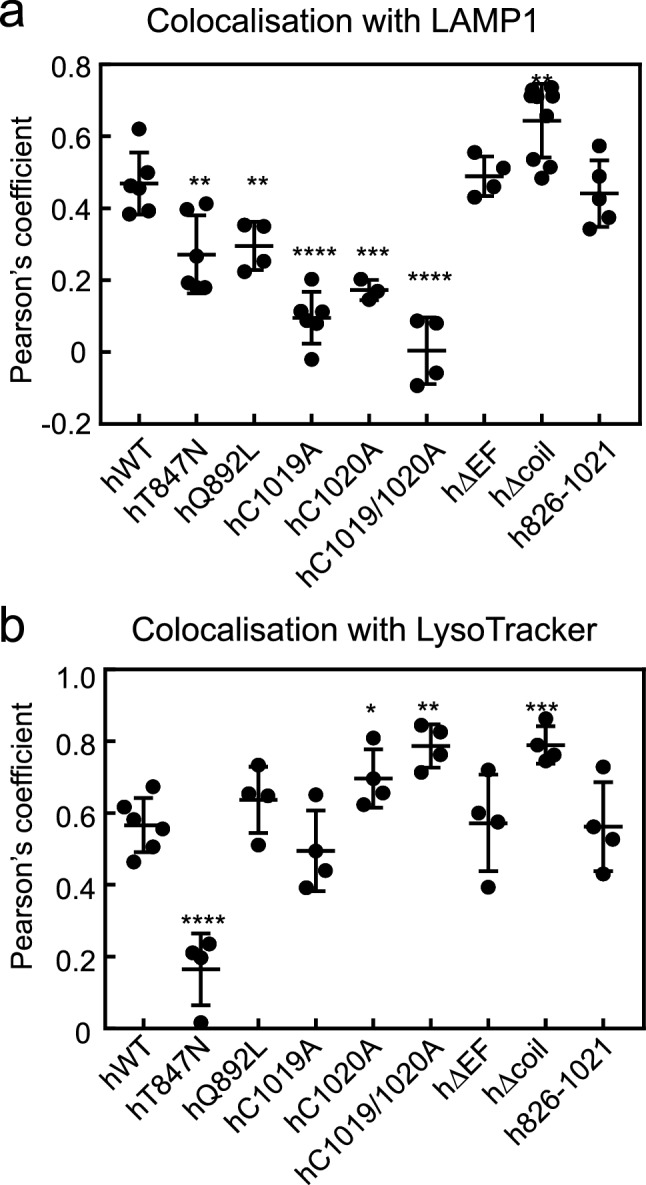
Comparing quantification of colocalisation of the hRab44 mutants with LAMP1 and LysoTracker. (**a**) Summary of quantitative analysis of colocalisation with LAMP1 (shown in Figs. 2, 3 and 4). (**b**) Quantitative analysis of colocalisation with LysoTracker. (shown in Fig. 5). The asterisks indicate statistical significance compared to the hWT, *P < 0.05, **P < 0.01, ***P < 0.001, or ****P < 0.0001.

Figure 6a shows a diagram summarizing quantitative analysis of colocalisation with LAMP1, supplementing the data shown in Figs. 2, 3 and 4. As compared with hWT, colocalisation with LAMP1 was significantly decreased for hT848N, hQ892L, hC1019A, hC1020A, and hC1019A/1020A mutants; significantly increased in the hΔcoil mutant; and unchanged in the hΔEF and h826-1021 mutants (Fig. 6a).

A quantitative analysis of colocalisation with LysoTracker is shown in Fig. 6b, supplementing the images in Fig. 5. When compared with the hWT, colocalisation with LysoTracker decreased only for the hT848N mutant, and increased for the hC1020A, hC1019/1020A, and hΔcoil mutants. The hQ892L, hC1019A, hΔEF and h826-1021 mutants colocalised with LysoTracker at similar levels as hWT (Fig. 6b). Of particular note, differences between observations with LAMP1 and LysoTracker colocalisation analysis were found in mutants that are diffusely localised, such as hQ892L, hC1019A, hC1020A, and hC1019/1020A, suggesting that LAMP1 and LysoTracker detect different subcellular compartments.

### Effects of Ca^2+^ modulators on localisation of human Rab44

**Figure 7:**
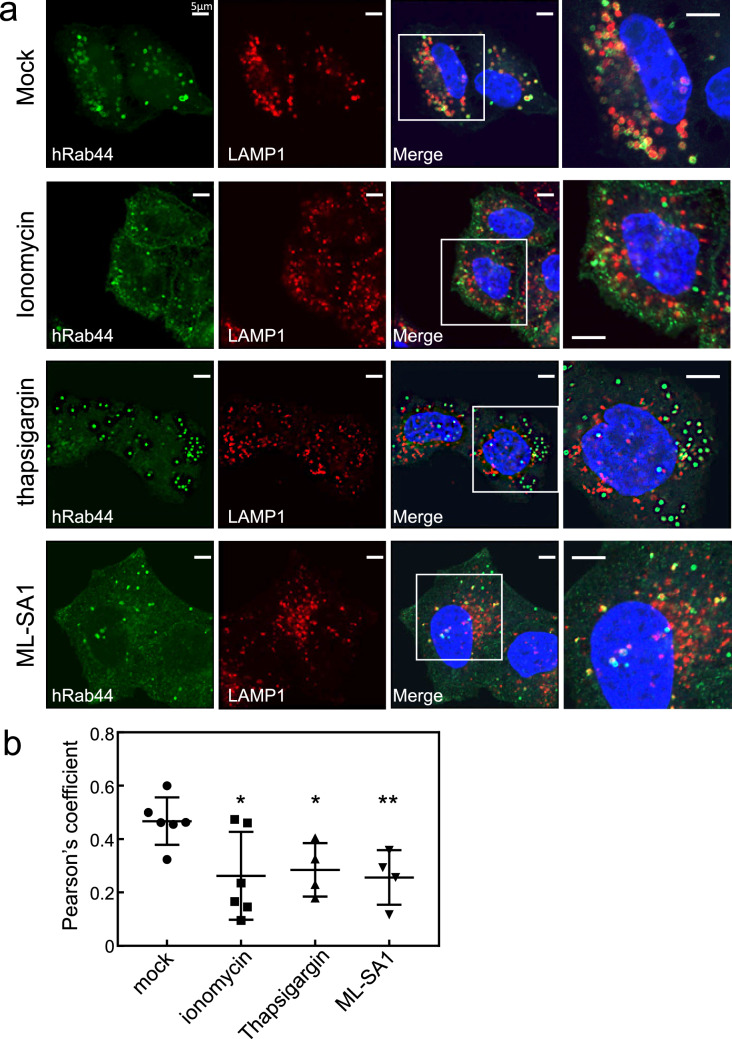
Effect of Ca^2+^ modulators on localisation of human Rab44 (hWT) expressed in HeLa cells**.** Human Rab44 (hWT)-expressing HeLa cells were treated with mock, ionomycin, thapsigargin, or ML-SA1. (**a**) Confocal laser microscopic analysis of HeLa cells immunofluorescently stained for LAMP1 (marker for late endosomes/lysosomes). Bar: 5 μm. (**b**) Quantitative analysis of the ratio of colocalisation of hWT with LAMP1.

Next, we assessed whether Ca^2+^ influx affects the subcellular localisation of human Rab44, since Rab44 encodes an EF-hand domain. As shown in Fig. 7, under basal conditions, hWT formed ring-like structures and partially surrounded the LAMP1-positive lysosomes in HeLa cells. However, when we treated hWT-expressing HeLa cells with ionomycin, a selective Ca^2+^ ionophore, the hWT partially translocated to small vesicles in the marginal region and to the plasma membrane and cytosol (Fig. 7a). We further examined the effects of other Ca^2+^-related reagents on the localisation of hWT. Upon treatment with thapsigargin, a Ca^2+^-ATPase inhibitor in the ER, hWT was detected in many vesicles distinct from LAMP1-positive lysosomes (Fig. 7a). ML-SA1 is a specific agonist for the lysosomal calcium channels transient receptor potential channel mucolipins (TRPML1–3). When hWT-expressing HeLa cells were treated with ML-SA1, the hWT localised to the plasma membrane and cytosol, and was also partially detectable in the lysosomes and non-lysosomal compartments (Fig. 7a). Following a quantitative analysis, we found that all the Ca^2+^ modulators significantly decreased colocalisation of hWT and LAMP1-positive lysosomes (Fig. 7b). These results indicate that transient Ca^2+^ influx induced by ionomycin or ML-SA1 causes partial translocation of hWT from the lysosomes to the plasma membrane and cytosol. In contrast, inhibition of intracellular Ca^2+^ with thapsigargin induces localisation of hWT to non-lysosomal vesicles.

### Effects of ionomycin-mediated Ca^2+^ influx on localisation of various human Rab44 mutants

**Figure 8:**
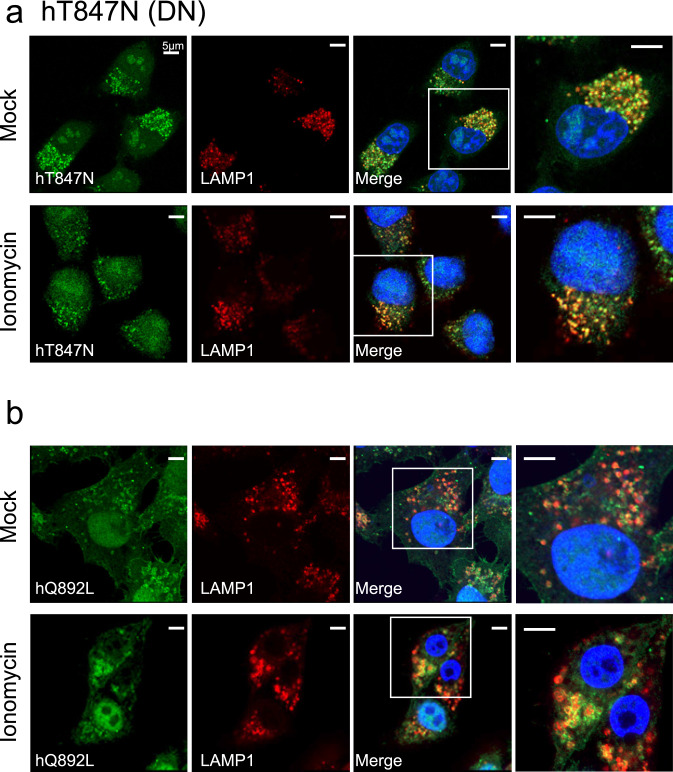

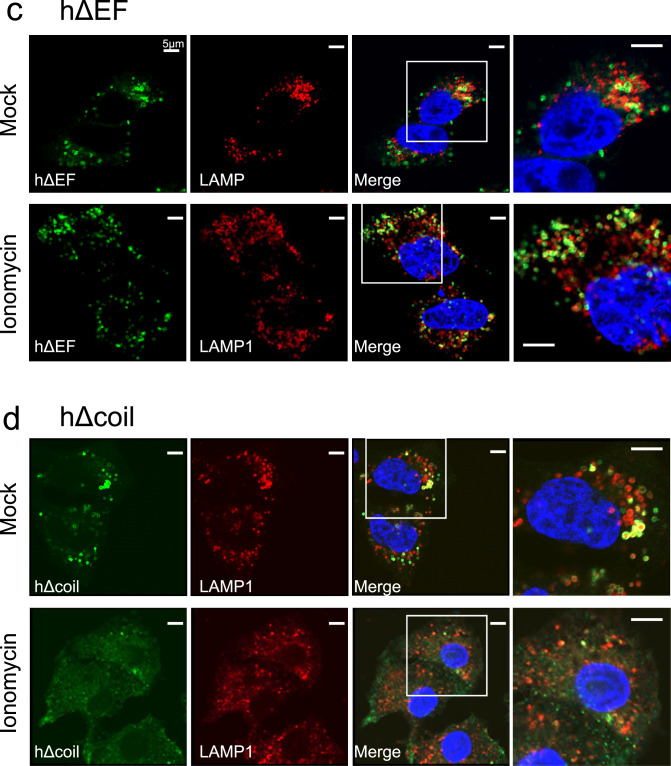

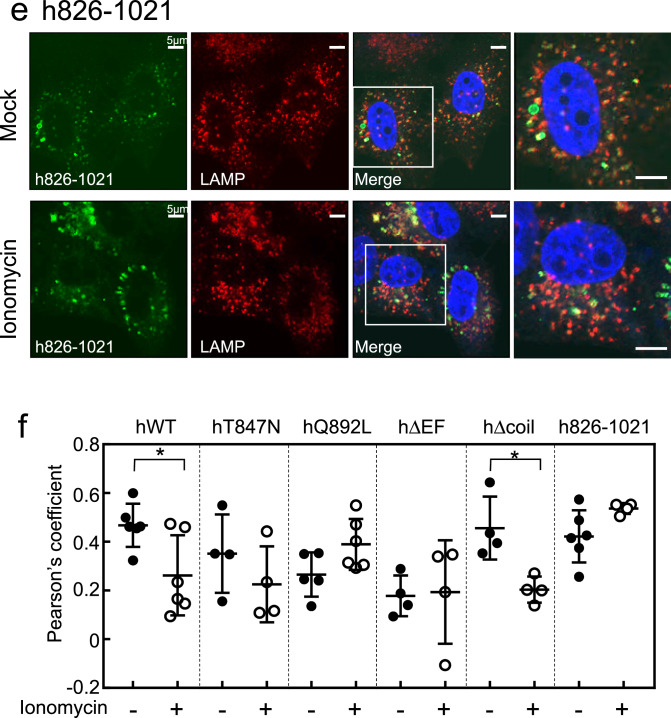
Effect of ionomycin on localisation of mutants of human Rab44 expressed in HeLa cells. **(a)** eGFP-hT847N (DN), (**b**) eGFP-hQ892L (CA), (**c**) eGFP-hΔEF, (**d**) eGFP-hΔcoil, and **(e)** eGFP-h826-1021 in HeLa cells. Human Rab44 (hWT)-expressing HeLa cells were treated with mock, or ionomycin. Confocal laser microscopic analysis of HeLa cells immunofluorescently stained for LAMP1 (marker for late endosomes/lysosomes). Bar: 5 μm. (**f**) Quantitative analysis of the colocalisation of the indicated Rab44 mutants with LAMP1 with or without ionomycin treatment.

We further assessed whether ionomycin-mediated Ca^2+^ influx affects the subcellular localisation of human Rab44 and its mutants. Localisation of the DN mutant hT847N was unaffected by ionomycin treatment, and was partially detectable both in the cytosol and with LAMP1-positive lysosomes (Fig. 8a). Without stimulation, the CA mutant hQ892L was detected in LAMP1-positive lysosomes surrounded by ring-like structures and partially in the plasma membrane (Fig. 8b). However, following ionomycin treatment, the formation of ring-like structures surrounding the LAMP1-positive lysosomes was slightly enhanced – though the difference was not statistically significant – and its localisation to the plasma membrane decreased (Fig. 8b). Importantly, the hΔEF mutant formed ring-like structures even after ionomycin treatment, and its localisation was nearly unchanged by stimulation (Fig. 8c). Therefore, the EF-hand domain is likely to be important for Ca^2+^-mediated localisation of human Rab44. The hΔcoil mutant localised partially to lysosomal and non-lysosomal vesicles without ionomycin, and was partially localised to the cytosol with ionomycin treatment (Fig. 8d). Ionomycin treatment significantly reduced the colocalisation ratio of the hΔcoil mutant with LAMP1-positive lysosomes (Fig. 8f). Ionomycin had no effect on the localisation of the h826-1021 mutant to non-lysosomal vesicles or its partial colocalisation with LAMP1-positive lysosomes (Fig. 8e). Quantification of the effects of ionomycin on colocalisation of Rab44 mutants with LAMP1-positive lysosomes is shown in Fig. 8f. Ionomycin treatment significantly reduced the colocalisation of hWT or hΔcoil mutant with LAMP1-positive lysosomes, but had little effect on the mutants lacking the EF-hand domain, such as the hΔEF and h826-1021 (Fig. 8f). Thus, the localisation of Rab44 is altered by ionomycin, and the EF-hand domain of human Rab44 is important for Ca^2+^-mediated regulation of Rab44 localisation.

## Discussion

In this study, we constructed various mutants of human Rab44 using site-directed mutagenesis. Wild-type human Rab44 (hWT) and the Rab44 mutant constructs were expressed exogenously in HeLa cells.

The expression level of the h826-1021 mutant was extremely low, for which there may be a few possible causes. First, whereas the wild-type protein contains 1021 amino acid residues, the h826-1021 is reduced to 19% of its original size with only 196 amino acid residues. Second, this fragment alone may be unstable as a protein. In other words, the N-terminal domains may be important for stable formation of the protein, even though the structure of this mutant is similar to that of other small Rab proteins.

Upon analysing the DN and CA mutants, Rab44 was found to share some common features with other small Rab GTPases. In many small Rab GTPases, differences in localisation have been observed between the DN and CA mutants. For example, when human Rab21 was overexpressed in HeLa cells, it predominantly localised to EEA1-containing early endosomes. The DN mutant Rab21(T33N) was concentrated in the perinuclear region, whereas the CA mutant Rab21(Q78L) localised to the reticular tubular network^20^. Therefore, it is possible that differences in localisation between the DN mutant hT847N and CA mutant hQ892L affect cell shape: hT847N caused a round cellular shape with clear contacts between cells, whereas hQ892L resulted in unclear cell–cell contact sites.

The lipidation-site mutants of Rab44 also shared some common features with those of the small Rab GTPases. The prenylatable cysteines are key factors for membrane targeting of Rab GTPases. For example, lipidation-site mutations in both Rab4 and Rab5 cause complete cytosolic localisation ^21^. Similarly, the lipidation-site mutant, hC1019A, was mostly localised to the cytosol and partially to lysosomes (Fig. 3c), whereas the lipidation-site mutants hC1020A and hC1019/1021A completely localised to the cytosol (Fig. 3d,e). Interestingly, hC1019A partially localised to LAMP1-positive lysosomes, whereas hC1020A was mostly diffusely distributed throughout the cytoplasm. Therefore, hC1020A appears to have a greater effect on subcellular localisation than hC1019A.

The EF-hand domain, which is known to be the Ca^2+^ binding site^22^, is likely to be required for the partial translocation of Rab44 into the plasma membrane and cytosol under conditions of transient Ca^2+^ influx. Consistent with this notion, transient Ca^2+^ influx caused partial translocation of the constructs containing the EF-hand domain, including hWT and hΔcoil, into the plasma membrane and cytosol. Considering that the CA mutant, hQ892L, partially localised to the plasma membrane and cytosol without stimulation, the EF-hand domain of Rab44 may be an important activation factor for translocation induced by Ca^2+^ mobilisation. In contrast, translocation of constructs lacking the EF-hand domain, including hΔEF and h826-1021 mutants, were virtually unaffected by ionomycin-mediated transient Ca^2+^ influx conditions. Taken together, the EF-hand domain of Rab44 is probably important for Ca^2+^-mediated translocation.

The coiled-coil domain appears to be important for localisation and formation of LysoTracker-positive vesicles as described below. Given that mutants lacking the coiled-coil domain, such as hΔcoil and h826-1021 mutants, had a higher colocalisation coefficient with LAMP1-positive lysosomes and a lower one with KDEL-positive compartment compared with hWT, it is likely that fusion of the mutants lacking the coiled-coil domain with lysosomes is increased but fusion with the ER is decreased. Generally, the coiled-coil domain-containing proteins have been proposed to function as tethering factors to target organelles prior to fusion or as scaffolds for the assembly of other factors important for fusion^23,24^. Therefore, it would be interesting to investigate the molecular mechanisms through which the Rab44 coiled-coil domain assists in localisation and organelle formation.

The size of LysoTracker-positive vesicles was affected by Rab44 expression and mutation. hWT induced larger vesicles compared to the control, the DN mutant hT847N induced smaller vesicles than the WT, and the CA mutant hQ892L induced larger vesicles than hWT; therefore, it is likely that Rab44 has effects on a large formation of LysoTracker-positive vesicles. Moreover, it is interesting that the combination of hC1020A and hC1019/1020A mutants and the combination of hΔcoil and h826-1021 mutants had the exact opposite effects on formation of LysoTracker-positive vesicles, although the mechanisms behind these effects remain unknown.

Interestingly, differences in colocalisation between LAMP1 and LysoTracker were observed in the mutants that are diffusely localised, such as hQ892L, hC1019A, hC1020A, and hC1019/1020A (Fig. 6). Specifically, these mutants had decreased colocalisation with LAMP1, and increased colocalisation with LysoTracker compared to hWT. There are known to be some differences between LAMP1-positive compartments and LysoTracker-stained organelles^25^. Therefore, we speculate that LysoTracker may detect LAMP1-negative acidic vesicles. Indeed, LysoTracker has reported to be an acidotropic dye that stains intermediate compartments, including autolysosomes, and phagolysosomes as well as late endosomes/lysosomes^26,27^.

Ionomycin- and ML-SA1-induced transient Ca^2+^ influx caused partial translocation of hWT from the lysosomes into the plasma membrane and cytosol. In contrast, inhibition of intracellular Ca^2+^ using thapsigargin induced translocation of hWT to non-lysosomal vesicles. Thus, the translocation of Rab44 is regulated by the intracellular Ca^2+^ level, and the EF-hand domain of Rab44 is important for the Ca^2+^-mediated translocation.

In conclusion, the roles of the EF-hand, coiled-coil domains, and lipidation sites in human Rab44 were analysed using deletion and point mutants. The EF-hand domain is required for partial translocation of Rab44 into the plasma membrane and cytosol under conditions of transient Ca^2+^ influx, and the coiled-coil domain is important for localisation, formation of LysoTracker-positive vesicles. The lipidation-site is essential for localisation to the membrane.

## Materials and methods

### Antibodies and reagents

Mouse monoclonal anti-GAPDH (Cat. No. M171-3), anti-GFP (Cat. No. 598), anti-EEA1 (Cat. No. M176-3), and anti-KDEL (Cat. No. M181-3) antibodies were purchased from Medical & Biological Laboratories (Nagoya, Japan). Mouse monoclonal anti-mouse LAMP1 (Cat. No. 555798) and anti-GM130 (Cat. No. 610823) antibodies were from BD Biosciences (Franklin Lakes, NJ, USA). Alexa Fluor 488-conjugated goat anti-rabbit IgG and Alexa Fluor 555-conjugated goat anti-mouse, anti-rat IgG, and anti-rabbit IgG were from ThermoFisher Scientific (Rockford, IL, USA). Ionomycin, thapsigargin, and ML-SA1 were purchased from FUJIFILM-WAKO (Osaka, Japan).

### Cell culture

HeLa cells were grown in Dulbecco’s modified Eagle’s medium (DMEM) containing 10% fetal bovine serum, 50 U/mL penicillin, and 50 μg/mL streptomycin at 37 °C in a 5% CO_2_ atmosphere.

### Retrovirus construction and expression of Rab44 and mutants

The full-length human *Rab44* gene was synthesised and cloned into pcDNA3.1 + N-DYK using GenScript (Piscataway, NJ, USA). The *Rab44* gene mutants were generated using polymerase chain reaction (PCR) using a PrimeSTAR mutagenesis kit (Takara, Shiga, Japan). The primers used for PCR were; for eGFP (for In-Fusion) Forward: GAATTAGATCTCTCGAGATGGTGAGCAAGGGCGAGGA and Reverse: TCTCTGTCCAGTCTCCTTGTACAGCTCGTC; Rab44 WT Forward: GGACGAGCTGTACAAGGAGACTGGACAGAGA, and Reverse: AATTCGTTAACCTCGAGTCAGGAGCAACAGCCG; hQ892L Forward: CAGCTGGCCTTGAGAGGTACCACAGTATG, and Reverse: TACCTCTCAAGGCCAGCTGTGTCCCAGAG; hT847N Forward: GGCAAAAACTCCTTCCTGCACCTGCTG, and Reverse: GAAGGAGTTTTTGCCCACGTTGGAGTC; hC1019A Forward: TTCGGCGCTTGCTCCTGACTCGAGGTTA, and Reverse: AGGAGCAAGCGCCGAATCTCTTGGGCG G; hC1020A Forward: GGCTGTGCTTCCTGACTCGAGGTTAAC, and Reverse: TCAGGAAGCACAGCCGAATCTCTTGGG; hC1019A/C1020A Forward: GGCGCTGCTTCC TGACTCGAGGTTAAC, and Reverse: TCAGGAAGCAGCGCCGAATCTCTTGGG; ΔEF Forward: CTCTCAGAGGAAGCCACTGCCCTCT, and Reverse: GGCTTCCTCTGAGAGGACCAAGACTC; Δcoil Forward: CTTCATGCAGCTACTGAGCAACTTT, and Reverse: GTAGCTGCATGAAGGCAAGGAACGC; and h826-1021 Forward: TGTACAAGCCCCAGGCCAACCCTGAT, and Reverse: GCCTGGGGCTTGTACAGCTCGTCCAT.

The PCR products were cloned into the retroviral vector, pMSCVpuro (Clontech, Mountain View, CA, USA), using an In-Fusion cloning kit (Clontech). The pMSCVpuro vector with an eGFP fragment insert yielding N-terminal eGFP-fusion proteins was kindly provided by Prof. Kosei Ito (Nagasaki University, Japan). The vectors were transfected into HEK293T cells using Lipofectamine 3000 (Life Technologies, Gaitherburg, MD, USA), according to the manufacturer’s instructions. After incubation at 37℃ in a 5% CO_2_ atmosphere for 48 h, the supernatants containing the viral particles were collected and used to infect HeLa cells. Cells stably expressing Rab44 were selected using puromycin (5 μg/mL) in the culture medium, and media was changed every third day after 3 weeks.

### Western blot analysis

Western blotting analysis was performed according to the protocol described previously^28–30^. Briefly, the cells were lysed in cell lysis buffer supplemented with protease inhibitors. Equal amounts of protein were subjected to sodium dodecyl sulphate polyacrylamide gel electrophoresis (SDS-PAGE) followed by transfer onto a polyvinylidene difluoride (PVDF) membrane. The blots were blocked with 5% milk in Tris-buffered saline (TBS) for 1 h at 25 °C, incubated with anti-GFP antibody (1:3000) for 2 h at 4 °C and washed four times with TBS containing 0.1% Tween 20. The blots were then incubated with horseradish peroxidase-conjugated secondary antibodies for 1 h at 25 °C, washed, and detected with Immobilon Forte ECL HRP substrate (Merck-Millipore, Burlington, MA, USA). Immunoreactive bands were analysed using a LAS-4000 Mini imaging system (Fujifilm, Tokyo, Japan). The membranes were reprobed with anti-GAPDH antibody, and analysed as above.

### Immunofluorescence microscopy

Cells were cultured on cover glasses and fixed with 4.0% paraformaldehyde in phosphate buffered saline (PBS) for 20 min at 25 °C. The fixed cells were then washed with PBS for 5 min twice, and permeabilised with 0.1% Triton X-100 in PBS for 15 min. The cells were blocked with 0.2% gelatin in PBS for 5 min, and subsequently incubated with primary antibodies for 1 h at 4 °C. The cells were washed with PBS-gelatin three times, and then incubated with the secondary antibody, Alexa Fluor 555-conjugated goat anti-mouse IgG. Nuclear staining was then performed using DAPI. The samples were subjected to microscopy using a laser-scanning confocal imaging system (LSM800; Carl Zeiss, AG, Jena, Germany) and analysed by Airyscan processing (ZEN2.3 software, Carl Zeiss). Subcellular distribution and colocalisation were quantified using Pearson's correlation coefficients. Quantitative data are presented as mean ± standard deviation (SD). Statistical analyses were performed using Prism 7 (GraphPad, San Diego, CA, USA). Unpaired *t*-tests were used to identify differences when a significant difference (*P < 0.05, **P < 0.01, ***P < 0.001, or ****P < 0.0001) was determined by analysis of variance.

### Live cell imaging

HeLa cells expressing the GFP-Rab44 constructs were grown on glass-bottom dish, and fluorescently labelled with 0.1 µM LysoTracker Red DND-99 (ThermoFisher) and 5 µg/mL Hoechst 33342 (Dojin) at 37 °C for 30 min. The cells were washed with media and observed under confocal microscopy. The area of LysoTracker-positive vesicles was calculated by employing ImageJ and subjected to statistical analysis.

### Ionomycin, thapsigargin, and ML-SA1 treatment

HeLa cells grown on cover glasses were incubated with 2 µM ionomycin, 10 µM thapsigargin, or 10 µM ML-SA1, for 15 min at 37 °C in a 5% CO_2_ atmosphere, and then immediately fixed with paraformaldehyde followed by immunofluorescence microscopy.

## Supplementary information

Supplementary Figure S1.
