# Supplementary material for: Role of the EF-hand and coiled-coil domains of human Rab44 in localisation and organelle formation
Source: Sci Rep. 2020 Nov 5;10:19149. doi: 10.1038/s41598-020-75897-7 (PMC7645795; doi:10.1038/s41598-020-75897-7)
Supplement: Supplementary file 1 — Supplementary Figure S1. [file 41598_2020_75897_MOESM1_ESM.pdf]

Supplementary Information

Role of the EF-hand and coiled-coil domains of human Rab44 in localisation and organelle formation

Kohei Ogawa[1, 2, 3], Tomoko Kadowaki[1], Mitsuko Tokuhisa[1, 2, 3], Yu Yamaguchi[2], Masahiro Umeda[3], and Takayuki Tsukuba[2]

[1]Department of Oral Life Science, Graduate School of Biomedical Sciences, Nagasaki University, Sakamoto 1-7-1, Nagasaki 852-8588, Japan.

[2]Department of Dental Pharmacology, Graduate School of Biomedical Sciences, Nagasaki University, Sakamoto 1-7-1, Nagasaki 852-8588, Japan.

[3]Department of Clinical Oral Oncology, Graduate School of Biomedical Sciences, Nagasaki University, Sakamoto 1-7-1, Nagasaki 852-8588, Japan.

Contents

Supplementary Figures

Figure S1: Original gel images of immunoblot analysis……………………………………3

Figuer 1 b

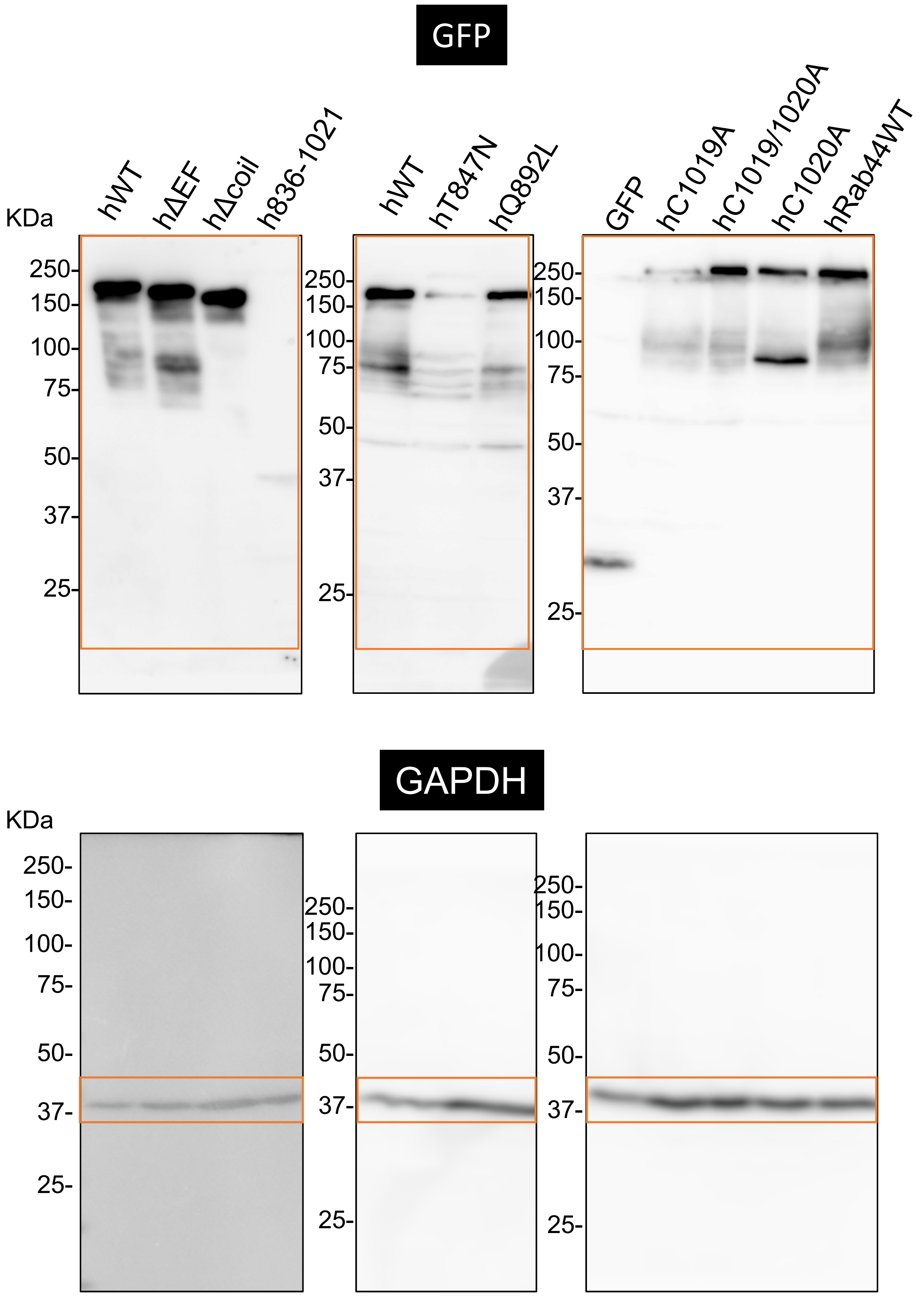

GFP
KDa
hWT
hΔEF
hΔcoil
h836-1021
hWT
hT847N
hQ892L
GFP
hC1019A
hC1019/1020A
hC1020A
hRab44WT
250-
150-
100-
75-
50-
37-
25-
GAPDH
KDa
250-
150-
100-
75-
50-
37-
25-
